# Analysis of argument structure constructions in a deep recurrent language model

**DOI:** 10.3389/fncom.2025.1474860

**Published:** 2025-06-16

**Authors:** Pegah Ramezani, Achim Schilling, Patrick Krauss

**Affiliations:** ^1^Department of English and American Studies, University Erlangen-Nuremberg, Erlangen, Germany; ^2^Cognitive Computational Neuroscience Group, Pattern Recognition Lab, University Erlangen-Nuremberg, Erlangen, Germany; ^3^Neuroscience Lab, University Hospital Erlangen, Erlangen, Germany

**Keywords:** cognitive computational neuroscience, argument structure constructions, linguistic constructions (CXs), recurrent neural networks (RNNs), LSTMs, sentence representation, computational linguistics, natural language processing (NLP)

## Abstract

Understanding how language and linguistic constructions are processed in the brain is a fundamental question in cognitive computational neuroscience. This study builds directly on our previous work analyzing Argument Structure Constructions (ASCs) in the BERT language model, extending the investigation to a simpler, brain-constrained architecture: a recurrent neural language model. Specifically, we explore the representation and processing of four ASCs–transitive, ditransitive, caused-motion, and resultative–in a Long Short-Term Memory (LSTM) network. We trained the LSTM on a custom GPT-4-generated dataset of 2,000 syntactically balanced sentences. We then analyzed the internal hidden layer activations using Multidimensional Scaling (MDS) and t-Distributed Stochastic Neighbor Embedding (t-SNE) to visualize sentence representations. The Generalized Discrimination Value (GDV) was calculated to quantify cluster separation. Our results show distinct clusters for the four ASCs across all hidden layers, with the strongest separation observed in the final layer. These findings are consistent with our earlier study based on a large language model and demonstrate that even relatively simple RNNs can form abstract, construction-level representations. This supports the hypothesis that hierarchical linguistic structure can emerge through prediction-based learning. In future work, we plan to compare these model-derived representations with neuroimaging data from continuous speech perception, further bridging computational and biological perspectives on language processing.

## Introduction

Understanding how language is processed and represented in the brain is a central challenge in cognitive neuroscience (Pulvermüller, 2002). In this paper, we adopt a usage-based constructionist approach to language, which views language as a system of form-meaning pairs (constructions) linking patterns to specific communicative functions (Goldberg, 2009, 2003). In particular, argument Structure Constructions (ASCs) such as transitive, ditransitive, caused-motion, and resultative constructions play a crucial role in language comprehension and production (Goldberg, 1995, 2006; Goldberg and Goldberg, 2019). These constructions are fundamental to syntactic theory and are integral to the way meaning is constructed in sentences. Investigating the neural and computational mechanisms underlying the processing of these constructions can provide significant insights into language and cognition (Pulvermüller, 2012, 2023; Pulvermüller et al., 2021; Henningsen-Schomers and Pulvermüller, 2022).

In recent years, advances in computational neuroscience have enabled the use of artificial neural networks to model various aspects of human cognition (Cohen et al., 2022). Furthermore, the synergy between AI and cognitive neuroscience has led to a better understanding of the brain's unique complexities (Krauss, 2024). AI models, inspired by neural networks (Hassabis et al., 2017), have allowed neuroscientists to delve deeper into the brain's workings, offering insights that were previously unattainable (Krauss, 2023). These models have been particularly useful in studying how different parts of the brain interact and process information (Savage, 2019). Among these neural network models, recurrent neural networks (RNNs) (Krauss et al., 2019b; Metzner and Krauss, 2022; Metzner et al., 2024), and specifically Long Short-Term Memory (LSTM) networks (Hochreiter and Schmidhuber, 1997), have shown considerable promise in modeling sequential data, such as natural language (Wang and Jiang, 2015).

Unlike transformer based large language models (Vaswani et al., 2017; Radford et al., 2018), which have gained popularity in natural language processing (NLP), LSTMs are designed to capture long-range dependencies in sequences without the need of a sliding window, making them more analogous to certain aspects of brain function related to temporal processing (Surendra et al., 2023).

In recent years, transformer-based architectures like BERT have dominated computational models of language processing, offering deep insights into the representation of Argument Structure Constructions (ASCs) (Ramezani et al., 2025). However, these models often lack constraints that mirror the structural and functional limitations of the human brain (Pulvermüller et al., 2021). In contrast, recurrent neural networks (RNNs), particularly Long Short-Term Memory (LSTM) networks, provide a more biologically plausible framework due to their sequential processing capabilities, which align more closely with temporal dynamics observed in neural activity. Our study builds upon previous analyses conducted with LSTMs, extending the investigation to transformer-based models to compare how different architectures represent ASCs. By contrasting the performance and internal representations of BERT and LSTM models, we aim to elucidate the extent to which each architecture captures the nuances of ASCs and their alignment with human linguistic processing.

This study employs a cognitive computational neuroscience approach (Kriegeskorte and Douglas, 2018). In particular, we explore how a deep recurrent language model, based on LSTM architecture, processes and represents different ASCs. We generated a custom dataset using GPT-4 (Radford et al., 2018, 2019; Brown et al., 2020), comprising 2000 sentences evenly distributed across four ASC types. By training the LSTM model on this dataset for next word prediction, we aim to examine how well the model distinguishes between the different constructions at various levels of its internal representations.

To analyze the internal activations of the LSTM model, we utilized dimensionality reduction techniques such as Multidimensional Scaling (MDS) (Torgerson, 1952) and t-Distributed Stochastic Neighbor Embedding (t-SNE) (Van der Maaten and Hinton, 2008) (cf. Methods). These techniques allow us to visualize high-dimensional data in a two-dimensional space, facilitating the identification of clusters corresponding to different ASCs. Additionally, we computed the Generalized Discrimination Value (GDV) (Schilling et al., 2021a) to quantify the clustering quality, providing an objective measure of how well the model's internal representations align with the different construction types (cf. Methods).

Our findings indicate that the LSTM model successfully differentiates between the four ASC types, with the most distinct clustering observed in the final hidden layer before the output. This suggests that even a relatively simple, brain-constrained recurrent neural network can capture complex syntactic structures. These results are in line with previous research demonstrating the emergence of word class and syntactic rule representations in recurrent language models.

In future work, we plan to extend this research by validating our findings using large language models such as BERT (Devlin et al., 2018; Krauss et al., 2024) and comparing the computational model's performance with neuroimaging data collected during continuous speech perception (Schilling et al., 2021b). By bridging the gap between computational models and neural data, we aim to advance our understanding of the neural mechanisms underlying language processing (Kriegeskorte and Douglas, 2018).

This study highlights the potential of recurrent neural language models to mirror linguistic processing in the human brain, offering valuable insights into the computational and neural mechanisms that underpin language understanding.

## Methods

###  Dataset creation using GPT4

To investigate the processing and representation of different Argument Structure Constructions (ASCs) in a recurrent neural language model, we created a custom dataset using GPT-4. This dataset was designed to include sentences that exemplify four distinct ASCs: transitive, ditransitive, caused-motion, and resultative constructions (cf. Table 1). Each ASC category consisted of 500 sentences, resulting in a total of 2000 sentences.

**Table 1:** Name, structure, and example of each construction.

**Constructions**	**Structure**	**Example**
Transitive	Subject + verb + object	The baker baked a cake.
Ditransitive	Subject + verb + object1 + object2	The teacher gave students homework.
Caused-Motion	Subject + verb + object + path	The cat chased the mouse into the garden.
Resultative	Subject + verb + object + state	The chef cut the cake into slices.

#### Selection of argument structure constructions

The four ASCs selected for this study are foundational to syntactic theory and represent different types of sentence structures:

Transitive Constructions: Sentences where a subject performs an action on a direct object (e.g., “The cat chased the mouse”).

Ditransitive Constructions: Sentences where a subject performs an action involving a direct object and an indirect object (e.g., “She gave him a book”).

Caused-motion Constructions: Sentences where a subject causes an object to move in a particular manner (e.g., “He pushed the cart into the garage”).

Resultative Constructions: Sentences where an action results in a change of state of the object (e.g., “She painted the wall red”).

#### Generation of sentences

To ensure the diversity and quality of the sentences in our dataset, we utilized GPT-4, a state-of-the-art language model developed by OpenAI (Radford et al., 2018, 2019; Brown et al., 2020). The generation process involved the following steps: Prompt Design: We created specific prompts for GPT-4 to generate sentences for each ASC category. These prompts included example sentences and detailed descriptions of the desired sentence structures to guide the model in generating appropriate constructions. Using the designed prompts, we generated 500 sentences for each ASC category. The generation process was carefully monitored to ensure that the sentences adhered to the syntactic patterns of their respective constructions.

### Manual review and filtering

The initial set of 2,000 sentences was generated using GPT-4, with 500 examples for each of the four Argument Structure Constructions (ASCs). At first, prompts were kept general (e.g., “Generate 500 transitive sentences”), resulting in syntactically and semantically appropriate sentences (e.g., She gave me her new dress, The teacher wrote his mother a letter). However, to enhance the experimental control and ensure a consistent sentence structure across ASC categories, we refined our prompts to enforce uniformity in word count and syntactic role length. For example, the refined prompt for the ditransitive construction was: “Generate 500 sentences with a ditransitive construction. Each sentence should maintain consistency in word count for each syntactic role. Example: The teacher gave students homework.” This prompt engineering approach yielded consistently structured sentences such as The manager offered employees bonuses and The baker made friends cupcakes. Once generated with this refined method, all sentences were manually reviewed and found to comply with the intended construction types, syntactic structure, and grammatical correctness. No sentences needed to be removed or corrected at this stage. Therefore, the final dataset required no manual edits or deletions after the controlled generation process, as all examples were valid according to the predefined syntactic templates.

#### Handling varying sentence lengths

Sentences in natural language vary in length, which poses a challenge for processing within neural networks. To address this, we used padding to standardize sentence lengths. Specifically, each sentence was padded to match the length of the longest sentence in the dataset. This padding ensures that all input sequences are of equal length, facilitating efficient batch processing during model training.

#### Text tokenization

To convert the textual data into a numerical format suitable for input into the neural network, we used a tokenizer. The tokenization process involved the following steps: Vocabulary Creation: Each unique word in the dataset was identified and assigned a specific ID number. This process resulted in a vocabulary list where each word corresponded to a unique integer identifier. Sentence Transformation: Each sentence was transformed into a sequence of these integer IDs, representing the words in the order they appeared. For instance, a sentence like “The cat chased the mouse” would be converted into a sequence of integers based on the IDs assigned to each word. By padding sentences to a uniform length and converting them into numerical sequences, we ensured that the dataset was ready for training the LSTM-based recurrent neural language model. These preprocessing steps are crucial for enabling the model to effectively learn and differentiate between the various ASCs.

#### Input representation: word IDs

To investigate how recurrent neural networks process Argument Structure Constructions (ASCs) independently of semantic information, we represented words using unique numerical IDs rather than pretrained embeddings. This means each word was mapped to a discrete integer value without any a priori encoding of semantic or syntactic similarity. The rationale behind this choice was to isolate the model's ability to learn constructional patterns based solely on syntactic structure and word position, rather than leveraging distributional semantics. This allowed us to assess whether ASC-specific internal representations could emerge in the network from purely sequential input and next-word prediction learning, reflecting syntactic processing in a controlled manner. While this approach does not model the full richness of semantic context as the brain does, it enables us to better study the emergence of structural differentiation akin to syntax.

Using word IDs instead of word embeddings in this study offers several advantages. Firstly, word IDs provide a simpler and more interpretable representation of the dataset, which aligns well with the study's focus on analyzing internal model activations and clustering of sentence representations based on Argument Structure Constructions (ASCs). This simplicity aids in isolating the effects of syntactic structures without the added complexity of pre-trained embeddings that carry semantic information from external contexts. Secondly, using word IDs ensures that the analysis remains focused on the syntactic and structural aspects of sentence processing, allowing for a clearer examination of how the LSTM model differentiates between different ASCs. This approach facilitates a more straightforward interpretation of the model's ability to capture syntactic patterns, which is the primary interest of this research.

The resulting dataset, comprising 2000 sentences represented as padded numerical sequences, serves as a robust foundation for training and analyzing the LSTM model. This carefully curated and preprocessed dataset enables us to investigate how different ASCs are processed and represented within the model, providing insights into the underlying computational mechanisms.

###  LSTM architecture

The LSTM model in this study is designed for next-word prediction without prior information about the type of sentence constructions. The initial goal is to evaluate the model's ability to predict the next word, while the main objective is to assess how well it understands and differentiates between the different constructions. The model architecture consists of four layers: Embedding Layer: This layer converts each sentence into a sequence of integer numbers, transforming the input words into dense vector representations. This step facilitates efficient processing by the LSTM layers. First LSTM Layer: This layer learns complex patterns and dependencies within the sequence of words, capturing the contextual information necessary for accurate next-word prediction. Second LSTM Layer: Building upon the first LSTM layer, this layer further refines the learned patterns and dependencies, enhancing the model's understanding of the sequence's structure. Dense Layer with Softmax Activation: The final layer outputs a probability distribution over all possible next words. The softmax activation function ensures that the output is a valid probability distribution, suitable for predicting the next word. The model ultimately outputs a one-hot vector, where the length corresponds to the number of possible next words, indicating the predicted probabilities for each word. This architecture enables the model to learn and represent the intricate patterns of different Argument Structure Constructions (ASCs), providing insights into how such constructions are processed and differentiated by a recurrent neural language model.

###  Analysis of hidden layer activations

After training the model, we assessed its ability to differentiate between the various constructions by analyzing the activations of its hidden layers. Given the high dimensionality of these activations, direct visual inspection is not feasible. To address this, we employed dimensionality reduction techniques to project the high-dimensional activations into a two-dimensional space. By combining different visualization and quantitative techniques, we were able to assess the model's internal representations and its ability to differentiate between the various linguistic constructions.

#### Multidimensional scaling (MDS)

This technique was used to reduce the dimensionality of the hidden layer activations, preserving the pairwise distances between points as much as possible in the lower-dimensional space. In particular, MDS is an efficient embedding technique to visualize high-dimensional point clouds by projecting them onto a 2-dimensional plane. Furthermore, MDS has the decisive advantage that it is parameter-free and all mutual distances of the points are preserved, thereby conserving both the global and local structure of the underlying data (Torgerson, 1952; Kruskal, 1964; Kruskal and Wish, 1978; Cox and Cox, 2008; Metzner et al., 2021, 2023a, 2022).

When interpreting patterns as points in high-dimensional space and dissimilarities between patterns as distances between corresponding points, MDS is an elegant method to visualize high-dimensional data. By color-coding each projected data point of a data set according to its label, the representation of the data can be visualized as a set of point clusters. For instance, MDS has already been applied to visualize for instance word class distributions of different linguistic corpora (Schilling et al., 2021b), hidden layer representations (embeddings) of artificial neural networks (Schilling et al., 2021a; Krauss et al., 2021), structure and dynamics of highly recurrent neural networks (Krauss et al., 2019a,b,c; Metzner et al., 2023b), or brain activity patterns assessed during e.g. pure tone or speech perception (Krauss et al., 2018a; Schilling et al., 2021b), or even during sleep (Krauss et al., 2018b; Traxdorf et al., 2019; Metzner et al., 2022, 2023a). In all these cases the apparent compactness and mutual overlap of the point clusters permits a qualitative assessment of how well the different classes separate.

#### t-Distributed Stochastic Neighbor Embedding (t-SNE)

This method further helped in visualizing the complex structures within the activations by emphasizing local similarities, allowing us to see the formation of clusters corresponding to different Argument Structure Constructions (ASCs). t-SNE is a frequently used method to generate low-dimensional embeddings of high-dimensional data (Maaten and Hinton, 2008). However, in t-SNE the resulting low-dimensional projections can be highly dependent on the detailed parameter settings (Wattenberg et al., 2016), sensitive to noise, and may not preserve, but rather often scramble the global structure in data (Vallejos, 2019; Moon et al., 2019). Here, we set the perplexity (number of next neighbors taken into account) to 100.

###  Generalized Discrimination Value (GDV)

To quantify the degree of clustering, we used the GDV as published and explained in detail in Schilling et al. (2021a). This GDV provides an objective measure of how well the hidden layer activations cluster according to the ASC types, offering insights into the model's internal representations. Briefly, we consider *N* points ***x*****_*n* = 1..*N*_** = (*x*_*n*, 1_, ⋯ , *x*_*n, D*_), distributed within *D*-dimensional space. A label *l*_*n*_ assigns each point to one of *L* distinct classes *C*_*l* = 1..*L*_. In order to become invariant against scaling and translation, each dimension is separately z-scored and, for later convenience, multiplied with $\frac{1}{2}$:


(1) $$\begin{array}{l} s_{n,d}=\frac{1}{2}\cdot \frac{x_{n,d}-\mu_{d}}{\sigma_{d}}. \end{array}$$

Here, $\mu_{d}=\frac{1}{N}\overset{N}{\underset{n=1}{\sum }}x_{n,d}$ denotes the mean,

and $\sigma_{d}=\sqrt{\frac{1}{N}\overset{N}{\underset{n=1}{\sum }}{\left(x_{n,d}-\mu_{d}\right)}^{2}}$ the standard deviation of dimension *d*.

Based on the re-scaled data points **s_*n*_** = (*s*_*n*, 1_, ⋯ , *s*_*n, D*_), we calculate the *mean intra-class distances* for each class *C*_*l*_


(2) $$\begin{array}{l} \bar{d}\left(C_{l}\right)=\frac{2}{N_{l}\left(N_{l}-1\right)}\overset{N_{l}-1}{\underset{i=1}{\sum }}\overset{N_{l}}{\underset{j=i+1}{\sum }}d\left({\text{s}}_{i}^{\left(l\right)},{\text{s}}_{j}^{\left(l\right)}\right), \end{array}$$

and the *mean inter-class distances* for each pair of classes *C*_*l*_ and *C*_*m*_


(3) $$\begin{array}{l} \bar{d}\left(C_{l},C_{m}\right)=\frac{1}{N_{l}N_{m}}\overset{N_{l}}{\underset{i=1}{\sum }}\overset{N_{m}}{\underset{j=1}{\sum }}d\left({\text{s}}_{i}^{\left(l\right)},{\text{s}}_{j}^{\left(m\right)}\right). \end{array}$$

Here, *N*_*k*_ is the number of points in class *k*, and ${\text{s}}_{i}^{\left(k\right)}$ is the *i*^*th*^ point of class *k*. The quantity *d*(**a, b**) is the euclidean distance between **a** and **b**. Finally, the Generalized Discrimination Value (GDV) is calculated from the mean intra-class and inter-class distances as follows:


(4) $$\begin{array}{l} \text{GDV}=\frac{1}{\sqrt{D}}\left[\frac{1}{L}\overset{L}{\underset{l=1}{\sum }}\bar{d}\left(C_{l}\right)-\frac{2}{L\left(L-1\right)}\overset{L-1}{\underset{l=1}{\sum }}\overset{L}{\underset{m=l+1}{\sum }}\bar{d}\left(C_{l},C_{m}\right)\right] \end{array}$$

whereas the factor $\frac{1}{\sqrt{D}}$ is introduced for dimensionality invariance of the GDV with *D* as the number of dimensions.

Note that the GDV is invariant with respect to a global scaling or shifting of the data (due to the z-scoring), and also invariant with respect to a permutation of the components in the *N*-dimensional data vectors (because the euclidean distance measure has this symmetry). The GDV is zero for completely overlapping, non-separated clusters, and it becomes more negative as the separation increases. A GDV of −1 signifies already a very strong separation.

###  Code implementation, computational resources, and programming libraries

All simulations were run on a standard personal computer. The evaluation software was based on Python 3.9.13 (Oliphant, 2007). For matrix operations the numpy-library (Van Der Walt et al., 2011) was used and data visualization was done using matplotlib (Hunter, 2007) and the seaborn library (Waskom, 2021). The dimensionality reduction through MDS and t-SNE was done using the sci-kit learn library.

The models were coded in Python. The neural networks were designed using the Keras (Chollet, 2015) and Keras-RL (Plappert, 2016) libraries. Mathematical operations were performed with numpy (Harris et al., 2020) and scikit-learn (Pedregosa et al., 2011) libraries. Visualizations were realized with matplotlib (Hunter, 2007) and networkX (Hagberg et al., 2008). For natural language processing we used SpaCy (Explosion, 2017).

## Results

To understand how the LSTM model differentiates between various Argument Structure Constructions (ASCs), we visualized the activations of its hidden layers using Multidimensional Scaling (MDS) and t-Distributed Stochastic Neighbor Embedding (t-SNE). Additionally, we quantified the degree of clustering using the Generalized Discrimination Value (GDV).

**Figure 1:**
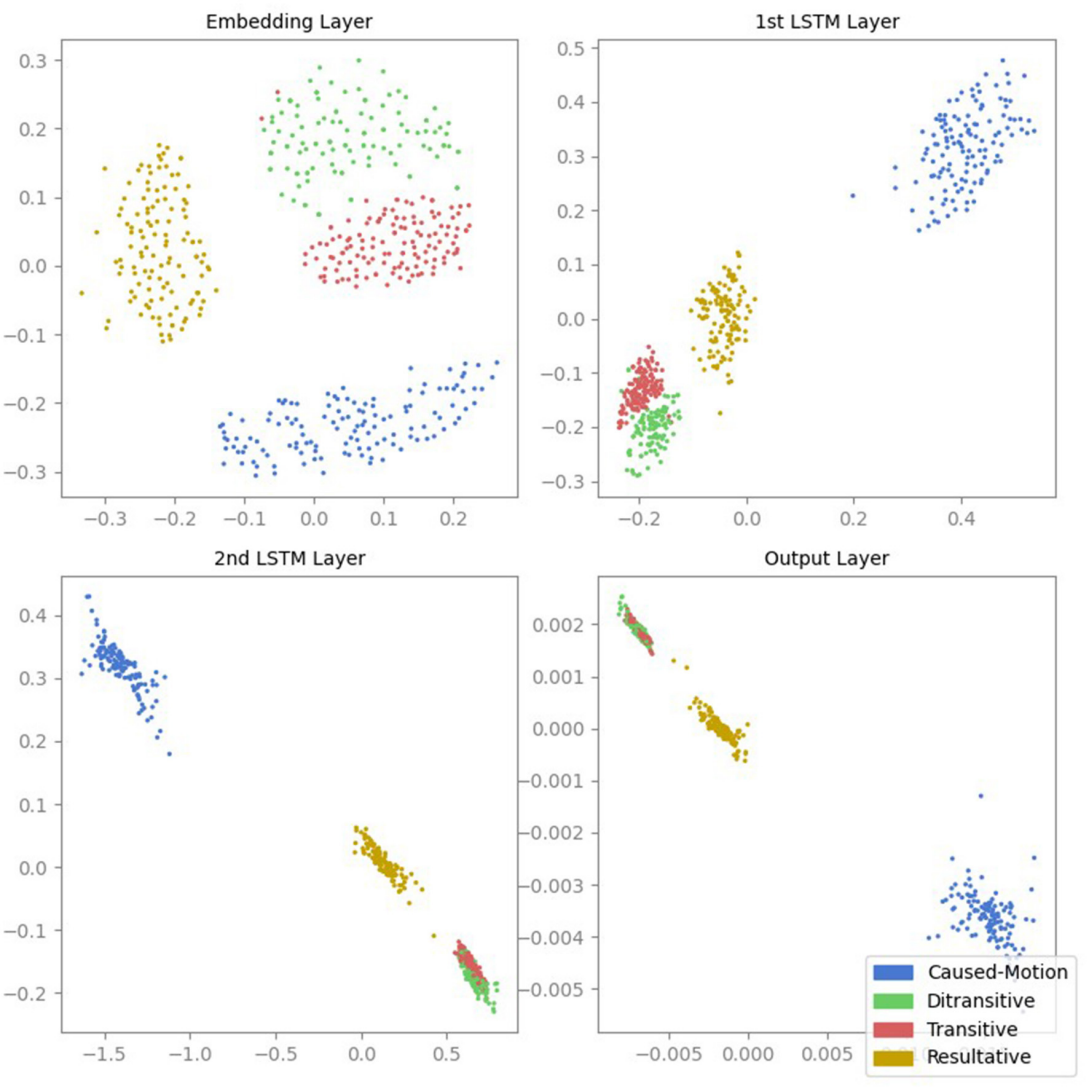
MDS projections of the activations from all four layers of the LSTM model. Each point represents the activation of a sentence, color-coded according to its ASC type: caused-motion (blue), ditransitive (green), transitive (red), and resultative (orange).

Figure 1 shows the MDS projections of the activations from all four layers of the LSTM model. Each point represents the activation of a sentence. The initial hidden layer already shows some separation between the different ASC types. As we move to the second LSTM layer, the separation between ASC types becomes more apparent, particularly with respect to the inter-cluster distances. However, the clusters for transitive and ditransitive sentences are closer to each other. In the third layer, the inter-cluster distances further increase, while the clusters for transitive and ditransitive sentences remain close to each other, indicating that the model is learning to differentiate between the ASCs more effectively and recognizes the similarity between transitive and ditransitive sentences. In the final output layer, the degree of clustering decreases slightly.

**Figure 2:**
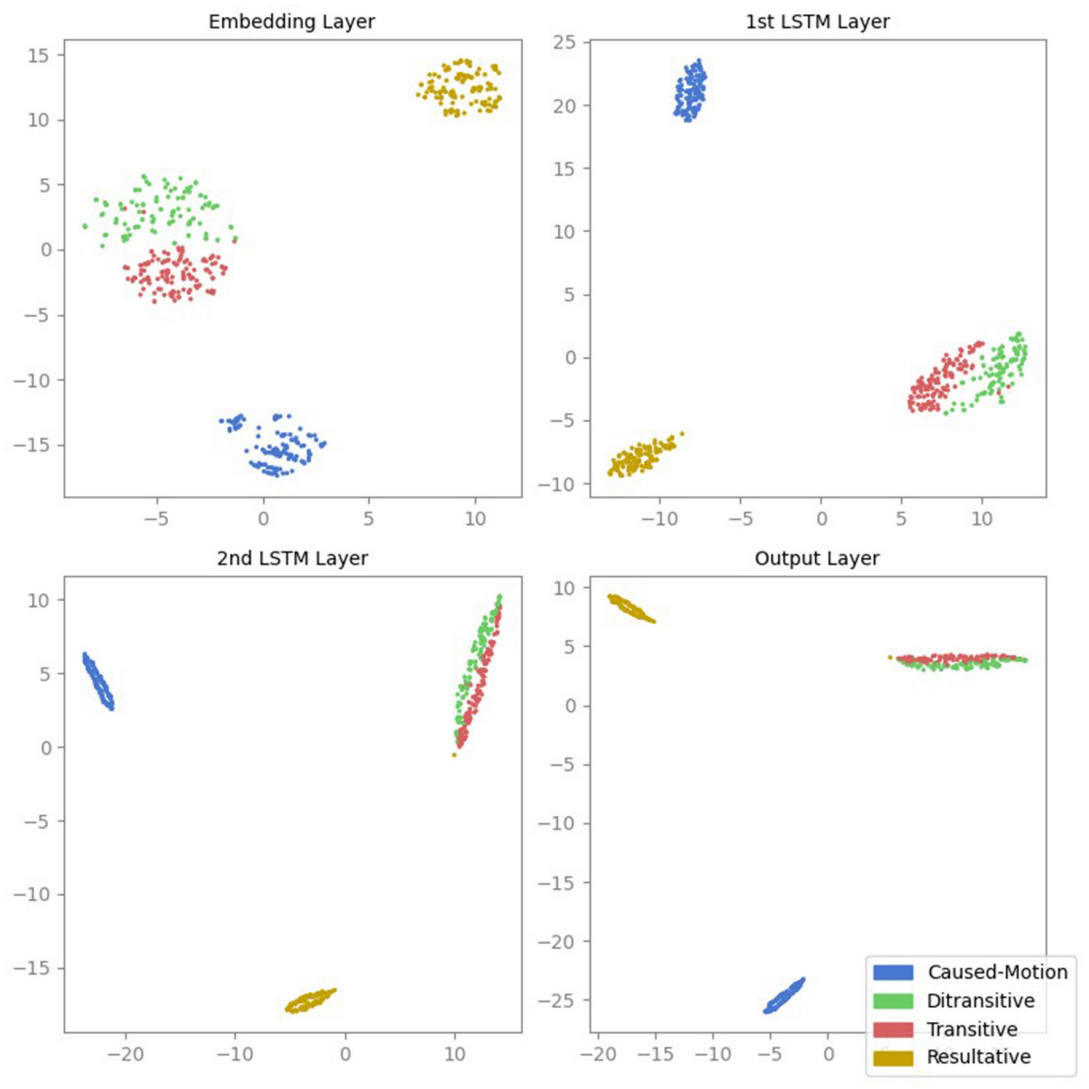
t-SNE projections of the activations from all four layers of the LSTM model. Each point represents the activation of a sentence, color-coded according to its ASC type: caused-motion (blue), ditransitive (green), transitive (red), and resultative (orange).

The corresponding t-SNE projections shown in Figure 2 yield qualitatively very similar results. The initial hidden layer shows some separation between ASC types, with increased and more apparent separation in the second layer, particularly in inter-cluster distances; this separation continues to improve in the third layer, while transitive and ditransitive sentences remain similar. The final layer shows a slight decrease in clustering degree.

**Figure 3:**
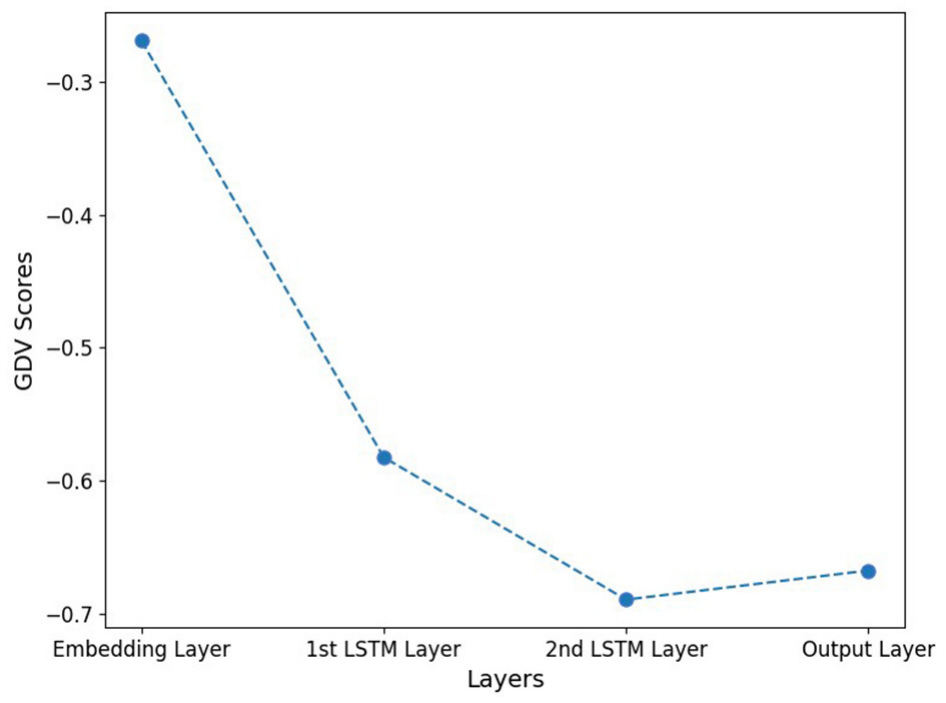
GDV score of hidden layer activations. Note that, lower GDV values indicate better-defined clusters. The qualitative results from the MDS and t-SNE projections are underpinned by the GDV with best clustering occurring in layer 3.

To quantitatively assess the clustering quality, we calculated the GDV for the activations of each hidden layer (cf. Figure 3). Lower GDV values indicate better defined clusters. The qualitative results of the MDS and t-SNE projections are supported by the GDV.

## Discussion

Our study aimed to understand how a recurrent neural language model (RNN) processes and represents different Argument Structure Constructions (ASCs) through the lens of cognitive computational neuroscience. Using a custom-generated dataset of sentences exemplifying four ASC types–transitive, ditransitive, caused-motion, and resultative–we trained an LSTM-based model for next-word prediction. The internal activations of the model's hidden layers were analyzed and visualized using Multidimensional Scaling (MDS) and t-Distributed Stochastic Neighbor Embedding (t-SNE), with clustering quality quantified by the Generalized Discrimination Value (GDV).

The dataset used in this study comprises 2,000 sentences evenly distributed across four Argument Structure Constructions (ASCs). While small by modern deep learning standards, this dataset was deliberately designed to be syntactically controlled and semantically minimal in order to isolate the effect of constructional variation. Each sentence follows a tightly specified structural template, ensuring that differences in internal representations can be attributed primarily to ASC type rather than lexical or semantic variability. This design enables a focused analysis of how recurrent networks abstract over syntactic patterns during prediction, without the confounds introduced by large-scale corpora. As such, the current work should be viewed as a first approximation–a proof-of-concept study establishing the feasibility of construction-based representation in LSTMs. Future studies will extend this approach using larger and more diverse corpora, including naturalistic input and richer semantic context.

Our analysis revealed that the model's sentence representations formed distinct clusters corresponding to the four ASCs in all hidden layers. This indicates the model's ability to differentiate between various syntactic structures. The clustering was most pronounced in the final hidden layer, just before the output layer. This suggests that as the information progresses through the layers, the model refines its understanding and separation of different ASCs.

The emergence of distinct ASC representations in our LSTM model aligns with previous studies that observed the formation of word class and syntax rule representations in recurrent language models trained on next-word prediction tasks (Surendra et al., 2023). This consistency across studies reinforces the idea that even relatively simple, brain-constrained neural network architectures (Pulvermüller, 2023) like LSTMs can capture complex syntactic structures inherent in natural language.

Our findings suggest that recurrent neural networks can serve as effective computational analogs for studying linguistic processing in the human brain. The ability of the LSTM model to differentiate between ASCs supports the notion that similar neural mechanisms might be at play in human language comprehension.

The pronounced clustering in the final hidden layer hints at a hierarchical processing structure, where initial layers capture basic features, and subsequent layers integrate and refine these features into more complex representations. This parallels theories of hierarchical processing in the human brain (Golestani, 2014; Badcock et al., 2019; Raut et al., 2020).

###  Limitations and future work

In recent years, transformer-based models like BERT have significantly advanced our understanding of Argument Structure Constructions (ASCs) in natural language processing. However, these models present certain limitations. For instance, studies have shown that transformers struggle with tasks requiring function composition and hierarchical structure processing, which are essential for accurately modeling complex linguistic patterns. Additionally, the high computational demands and resource-intensive nature of training transformer models pose practical challenges. Furthermore, while BERT has demonstrated the ability to capture ASC representations, there is a need to explore how models with different architectures, particularly those that are more biologically plausible, process these constructions (Pulvermüller et al., 2021). Our study addresses these gaps by investigating how a recurrent neural network (LSTM) represents and processes ASCs, offering insights into alternative modeling approaches that may overcome some of the limitations associated with transformer-based models.

Our custom dataset, while carefully generated and balanced, is limited to 2000 sentences and four specific ASCs. Future studies could expand the dataset to include a wider variety of constructions and larger sentence pools to ensure generalizability.

Furthermore, our model used word IDs instead of embeddings, focusing on syntactic structures without semantic information. Incorporating pre-trained word embeddings (Almeida and Xexéo, 2019) in future studies could provide a more holistic view of how semantic and syntactic information interact in neural representations.

While our computational findings are promising, they need to be validated against empirical neuroimaging data. Comparing the LSTM's internal representations with brain activation patterns during continuous speech perception (Schilling et al., 2021b; Schüller et al., 2023; Garibyan et al., 2022) could provide deeper insights into the neural correlates of ASC processing. Techniques like EEG and MEG could be used to collect neural data during language tasks (Schüller et al., 2024), enabling a direct comparison with the model's activations using techniques such as representational similarity analysis (Kriegeskorte et al., 2008). This would help bridge the gap between computational models and real-world brain function (Meeter et al., 2007; Kriegeskorte and Douglas, 2018).

Although LSTMs are effective, they represent an earlier generation of neural network architectures (Hochreiter and Schmidhuber, 1997). Exploring more advanced models, such as transformers (Vaswani et al., 2017), could provide additional insights into the processing and representation of ASCs (Goldberg, 1995, 2006). Transformers, with their attention mechanisms, might offer a more nuanced understanding of how different constructions are represented and processed, potentially revealing more about the interaction between different levels of linguistic information.

While MDS and t-SNE are valuable tools for visualizing internal representations of high-dimensional neural activations, they also introduce potential artifacts. MDS is parameter-free and preserves global pairwise distances, but dimensionality reduction can still distort relationships when projecting from hundreds of dimensions to two. t-SNE, conversely, focuses on preserving local neighborhood structure but often exaggerates inter-cluster separations and is sensitive to hyperparameter choices (e.g., perplexity). To ensure the robustness of our findings, we used both methods in parallel and verified that the observed clustering patterns were consistent across projections. More importantly, we calculated the Generalized Discrimination Value (GDV) directly in the original high-dimensional space, providing a quantitative measure of class separability unaffected by projection. This combination of techniques allowed us to validate the existence of construction-specific structure in the hidden layers beyond the limitations of any single method.

While our results show that a recurrent neural network trained on syntactically controlled input can develop internal representations that distinguish between different Argument Structure Constructions (ASCs), it is important to explicitly acknowledge the theoretical and neurobiological limitations of the modeling approach employed. Long Short-Term Memory (LSTM) networks, though widely used in cognitive modeling, diverge in several critical ways from the neural mechanisms underlying language processing in the human brain. LSTMs process input in discrete time steps and store information via artificial gating mechanisms, which – though functionally useful – are not based on known neurophysiological processes. Unlike biological systems, LSTMs do not operate through distributed population codes, spiking dynamics, or anatomically grounded connectivity patterns. Moreover, the architecture does not inherently support hierarchical compositionality, incremental parsing strategies, or top-down predictive mechanisms that are central to contemporary neuro-cognitive models of language comprehension.

For these reasons, we do not interpret our results as a biologically faithful account of how ASCs are processed in the human brain. Instead, our aim was to explore whether meaningful structural differentiation – akin to grammatical construction types– can emerge from sequence learning alone under carefully controlled input conditions. The choice to use LSTM, rather than more biologically plausible models, was motivated by a desire for architectural simplicity, interpretability, and compatibility with prior work in both natural language processing and cognitive modeling. We see this study as a computationally constrained, first-step analysis designed to isolate construction-specific representations in the absence of semantic and contextual confounds. Importantly, this approach enables hypothesis generation regarding which features of linguistic structure are likely to emerge under predictive pressure and which may require stronger inductive biases or interaction with semantic knowledge.

Future work will aim to bridge this gap more directly by integrating human data into the modeling framework. Specifically, we plan to conduct cross-validation using empirical benchmarks such as EEG or MEG data collected during sentence comprehension tasks involving argument structure variation. Techniques like representational similarity analysis (RSA) can then be used to compare neural activation patterns with model-internal representations across time. Such efforts will allow us to evaluate the extent to which recurrent or transformer-based models approximate human linguistic processing at the algorithmic and representational levels. Ultimately, we believe that meaningful progress in cognitive computational neuroscience requires this kind of hybrid methodology – where computational models are not only interpretable but also grounded in empirical data from the brain. Our current findings offer a stepping stone toward that goal by showing that construction-specific representations can emerge even in relatively simple models trained under syntactic constraints.

## Conclusion

Our study demonstrates that even a relatively simple LSTM-based recurrent neural network can effectively differentiate between various Argument Structure Constructions, mirroring some aspects of human linguistic processing. The distinct clustering of sentence representations suggests that the model captures essential syntactic structures, supporting its use as a computational tool in cognitive neuroscience. Future work should aim to validate these findings with larger datasets and neuroimaging data, and explore the capabilities of more advanced neural network architectures. By doing so, we can further our understanding of the computational and neural mechanisms underlying cognition and language processing in brains, minds and machines (Tuckute et al., 2024; Schilling et al., 2023).

## Data Availability

The raw data supporting the conclusions of this article will be made available by the authors, without undue reservation.
